# Improving Patients’ Sleep in an Acute Mental Health Ward Using Non-Pharmacological Interventions

**DOI:** 10.1192/bjo.2025.10393

**Published:** 2025-06-20

**Authors:** Theodoros Mavrogiannidis, Ghada Alshehabi, Alessandro Malfatto

**Affiliations:** Central and North West London NHS Foundation Trust, London, United Kingdom

## Abstract

**Aims:** Interruption of sleep-wake behavioural patterns and circadian rhythms has been associated with the development and worsening of a range of mental health disorders, including depression, bipolar disorder, and schizophrenia, and specific high-risk outcomes such as aggression and suicidality. In full knowledge of the above, we aimed to improve patients’ self-reported sleep quality in an acute male ward, by 20% by the end of January 2025.

**Methods:** An initial survey was conducted for patients to rate their sleep quality on a Numeric Rating Scale (1–10, where 1 = a worst night sleep and 10 = a best night sleep). This survey included close- and open-ended questions for patients to identify perceived barriers to good sleep. Responses were collected over one week from all consenting patients on the ward (10/18 patients). Insights from the survey were used to design targeted interventions addressing the key contributors to poor sleep. These interventions included: a) Offering earplugs to patients; b) Posters with QR codes for a free white noise app to mask disruptive noises; c) Sleep hygiene education through leaflets, with practical tips to improve sleep. A following survey was conducted after two weeks to measure the results of our interventions.

**Results:** Initial survey results included: a) 6/10 median sleep rating reported by our patients, pre-intervention; b) 5/10 of our patients reported their sleep to be disturbed by noise on the ward; c) none of our patients reported sleep to be disturbed by the temperature or lighting of the room; d) 2/10 reported psychiatric symptoms such as auditory hallucinations to disturb their sleep. Results after interventions included: a) all of our patients stated that they received the sleep hygiene booklet, were counselled about the tips, and saw the posters around the ward; b) 11/16 included in the post-intervention survey reported that they found the tips useful; c) 10/16 had used the earplugs and 7/10 of these had found them helpful; d) 1/16 downloaded and used the white noise app; e) 7/10 median sleep rating was reported post-intervention.

**Conclusion:** Non-pharmacological interventions such as earplugs and sleep hygiene education proved to be effective in improving patients’ quality of sleep. The development of a standardized protocol that includes these sleep-friendly practices has been implemented on the ward. Methods’ limitations such as baseline sleep medications and the complexity of contributing factors were taken into consideration.

